# Determination of the Available Energy of Corn DDGS Fed to Pregnant Sows

**DOI:** 10.3390/ani15162370

**Published:** 2025-08-12

**Authors:** Can Zhang, Bo Cheng, Lei Xue, Ling Liu, Fenglai Wang, Jianjun Zang

**Affiliations:** State Key Laboratory of Animal Nutrition and Feeding, College of Animal Science and Technology, China Agricultural University, No. 2 Yuanmingyuan West Road, Beijing 100193, China; b20233040380@cau.edu.cn (C.Z.);

**Keywords:** Corn DDGSs, pregnant sows, substitution level, available energy

## Abstract

Based on an established appropriate substitution level for corn distillers dried grains with solubles (DDGSs) replacing energy-supplying components in the basal diet for pregnant sows, the substitution method was employed to determine the available energy values of corn DDGSs. In Exp. 1, forty pregnant sows (gestation day = 50 ± 5 d; body weight = 220 ± 24.9 kg; parity, 4 to 6) were randomly allocated into five treatments. The control group was fed a corn–soybean basal diet, while test diets contained 20%, 30%, 40%, 50% corn DDGSs replacing the energy-supplying portion of the basal diet. In Exp. 2, the available energy of five corn DDGS sources was determined using the substitution method at a 30% substitution level. Twelve pregnant sows (gestation day = 50 ± 5 d; body weight = 225.4 ± 29.2 kg; parity, 4 to 6) were arranged in a 6 × 3 Youden square design. Dietary treatments consisted of the corn–soybean basic diet and five test diets incorporating different corn DDGS types. Increasing the substitution level of corn DDGSs displayed both linear and quadratic effects (*p* < 0.05) on the apparent total tract digestibility (ATTD) of dry matter (DM), organic matter (OM), acid detergent fiber (ADF), ether extract (EE) and gross energy (GE) in diets. The ATTD of neutral detergent fiber (NDF), digestible energy (DE) and metabolizable energy (ME) was quadratically affected by different substitution levels (*p* < 0.05), with the highest value achieved at the 30% substitution level. As the substitution level of corn DDGSs increased, the ATTD of ADF in corn DDGSs decreased. In contrast, the ATTD of crude protein (CP) increased either linearly or quadratically (*p* < 0.05), and a significant quadratic effect was observed for the ATTD of EE in corn DDGSs (*p* < 0.05). Although the GE, DE, and ME of corn DDGSs were not significantly influenced by the substitution level, the 30% corn DDGSs resulted in the lowest coefficients of variation (CV). Therefore, a 30% substitution level of corn DDGSs is recommended for energy-supplying components in the basal diet of pregnant sows using the substitution method. The ranges of DE, ME and net energy (NE) of five corn DDGSs samples were 15.58–18.07, 12.17–16.42 and 8.76–15.88 MJ/kg DM, respectively. The absolute value of relative error (|RE|) between the predicted available energy values obtained from the prediction equations established in Exp. 2 and the determined values were below 5%, except for ME for corn DDGSs N3 (5.81%).

## Simple Summary

Corn distiller’s dried grains with solubles (DDGSs) are a highly attractive alternative to corn and soybean meal in animal feeds. Accurately determining the net energy (NE) value of corn DDGSs is essential for formulating precise diets for pregnant sows. The substitution method has been widely employed to determine the available energy value of feed ingredients that cannot be fed directly to pigs. However, the appropriate substitution level of corn DDGSs in the test diets of the substitution method, which influences the accuracy of the assessed energy, and its corresponding net energy in pregnant sows remain unclear. Therefore, this study aims to establish the appropriate substitution level of corn DDGSs in test diets relative to the energy-supplying portion of the basal diet, and to accurately measure the digestive energy, metabolizable energy and NE values of different corn DDGSs types in pregnant sows.

## 1. Introduction

Corn distillers’ dried grains with solubles (DDGSs), a byproduct of corn ethanol production, serves as a viable protein and energy alternative to soybean and corn in animal feeds. Research has demonstrated that including a specific quantity of corn DDGSs in animal diets does not adversely affect the performance of livestock and poultry [1]. Nevertheless, its nutritional composition and available energy vary significantly with corn sources, processing technique (dry-grind and wet-milling), and oil extraction practices [1]. Precise characterization of the nutritional values of corn DDGSs for swine is therefore crucial for effective precision feeding management, which can reduce feed costs, improve utilization efficiency, and minimize environmental pollution [2]. Given that energy accounts for more than 50% of total feed costs in commercial operations [3], accurate determination of the available energy content of this widely used feed ingredient is essential.

The previous research has focused on determining the digestible energy (DE) and metabolizable energy (ME) values of corn DDGSs in growing pigs [4,5,6], with limited data available for pregnant sows. Moreover, the net energy (NE) value of corn DDGSs for sows has not been reported. Consequently, a comprehensive investigation into the available energy of corn DDGSs in pregnant sows is necessary. Various methods can be employed to determine the available energy of ingredients, including direct method, substitution method, regression method and predictive equation method [7]. Typically, two or more of these methods are used in combination to evaluate the available energy. The substitution method is especially suitable for determining the available energy of ingredients that cannot be directly fed to pigs. Due to potential mycotoxin contamination and palatability issues, corn DDGSs cannot be used as a sole feed ingredient. Consequently, the substitution method is an appropriate approach for determining the available energy concentrations of corn DDGSs. The substitution level of the test ingredient is a critical factor influencing the accuracy of available energy determination via the substitution method [7]. While higher substitution levels theoretically enhance precision, excessive inclusion may introduce significant nutritional disparities between basal and test diets, and such compositional differences can compromise the accuracy of available energy assessment for the test ingredient [8].

Therefore, the objective of this study was to determine the appropriate level of corn DDGSs in the test diet, replacing the energy-supplying components in the basal diet for pregnant sows using the substitution method, and subsequently evaluate the available energy concentration of diverse corn DDGSs sources in pregnant sows.

## 2. Materials and Methods

All procedures used in the animal experiments were authorized and approved by the Institutional Animal Care and Use Committee of China Agricultural University (Beijing, China; AW10304202-1-1). The experiments were conducted at the Fengning Swine Research Unit of China Agricultural University located in Chengde, Hebei, China.

### 2.1. Corn DDGS Samples

In order to minimize the impact of interactions among various ingredients on the determination of available energy in corn DDGSs, Stein [9] demonstrated that ME of high-oil corn DDGSs for growing pigs is comparable to that of corn. When high-oil corn DDGSs in test diet are chosen to substitute for energy-supplying components (corn and soybean meal) in basal diet, the nutritional levels of each diet can be roughly equal, thereby reducing confounding effects of dietary matrix difference on the available energy assessment. Therefore, high-oil corn DDGSs (12.4% EE) sourced from Hefeng Pastoral Industry (Shenyang, China) were selected for Exp. 1. The vitamin and mineral premix fed to pregnant sows was provided by Beijing Tonglixingke Agricultural Technology Company Limited (Beijing, China). In Exp. 2, five corn DDGSs samples with varying ether extract (EE) content were selected from different regional suppliers in China: J1: Heilongjiang Shenglong Industrial Co., Ltd. (Harbin, China); J2: Heilongjiang Zhongke Green Biotechnology Co., Ltd. (Mudanjiang, China); N3: Henan Mengzhou Huaxing Co., Ltd. (Jiaozuo, China); X4: Xinjiang Bosheng Liquor Brewing Co., Ltd. (Bortala Mongolian Autonomous Prefecture, China); and C5: Chifeng Ruiyang Chemical Co., Ltd. (Chifeng, China). All samples were classified according to the classification criteria for corn DDGSs as outlined by NRC (2012) [10]. Specifically, J1 was classified as low-oil DDGSs, X4 as medium-oil DDGSs, while J2, N3, and C5 were categorized as high-oil corn DDGSs. Analyzed nutrient concentrations of each corn DDGSs were presented in Table 1 for Exp. 1 and Exp. 2.

### 2.2. Animals, Diets and Experimental Design

In Exp. 1, based on their initial body weight (220.0 ± 24.9 kg) and parity (4–6 parities), 40 pregnant sows (Landrace × Large White; gestation day = 50 ± 5 d) were randomly allocated to 5 treatments, with 8 replicates per group, and 1 pregnant sow per replicate. The five treatment groups were: (1) basal diet (Basal-1); (2) Basal-1 + 20% corn DDGSs (D20); (3) Basal-1 + 30% corn DDGSs (D30); (4) Basal-1 + 40% corn DDGSs (D40); (5) Basal-1 + 50% corn DDGSs (D50). Chromic oxide (Cr_2_O_3_, analytical grade, purchased from Shanghai Aladdin Biochemical Technology Co., Ltd., Shanghai, China) was added at 0.30% (*w*/*w*) to all diets as an indigestible marker to determine nutrient digestibility. Each pregnant sow was housed individually in a stall (2.2 m × 0.6 m × 0.9 m) with a partially slatted floor. The house was maintained at an average temperature of 22 ± 2 °C and humidity of 50–60%. Exp. 1 comprised two consecutive periods, each comprising a 7-day adaptation period (days 1–7) followed by a 5-day collection period NRC (2012). The authors of [10] recommended daily feed intake of 2.21 kg for sows in mid-gestation sows. Sows had (days 8–12) [10]. Pregnant sows were fed three times daily at 05:30, 10:30, and 15:30. Daily feed allowance was determined by the sows’ backfat thickness: 2.2 kg/day for sow with backfat thickness exceeding 15 mm; 2.5 kg/day for those with backfat thickness of 15 mm or less. These amounts were in line with or slightly higher than the free access to water throughout the experiment.

In Exp. 2, twelve pregnant sows (Landrace ×Large White; gestation day = 50 ± 5 d) were selected based on initial weight (225.4 ± 29.2 kg) and parity (4–6). Dietary treatments consisted of a basal diet (Basal-2) and 5 test diets containing 30% corn DDGSs from 5 different sources (J1-D1, J2-D2, N3-D3, X4-D4, C5-D5). In Exp. 1 and Exp. 2, all ingredients were thoroughly mixed for 5 min using a twin-shaft paddle mixer (Model SLH-500, Muyang Group, Yangzhou, China) to prepare the mash diets. Due to the availability of 6 open-circuit respiration chambers, twelve sows were divided evenly into two groups and transferred to the open-circuit respiration chamber in an alternating manner. A replicated 6 × 3 Youden square design was employed with 6 diets and 3 successive periods. Each Youden square included 6 sows and 6 respiration chambers, resulting in 6 replicates per treatment. Exp. 1 showed that chromium first appeared in the feces on the 4th day, confirming that the experimental diet had begun to be excreted by that time. Guided by this result and previous studies [11,12], Experiment 2 was conducted so that each period lasted for 10 days (6-day dietary adaptation followed by a 4-day heat production (HP) measurement period. During the first five days (d 1 to 5), pregnant sows were fed in metabolism crates (1.7 m × 0.7 m × 1.4 m) at the temperature of 22 ± 2 °C. On the morning of day 6, sows were transferred to the chambers for the measurement of O_2_, CO_2_ and CH_4_ concentrations. On day 9, pregnant sows were fasted, heat production (HP) measured from 22:30 (d 9) to 06:30 (d 10) was designed as the fasting heat production (FHP). To allow a consistent time span for comparing FHP to total HP, the 8 h HP was extrapolated to a 24 h period [13]. The pregnant sows were fed equal-sized meals twice a day at 08:30 and 15:30. Sows were fed at 544 kJ ME/kg BW^0.75^ d^−1^ [14], which was approximately 1.3 times the maintenance energy requirement for pregnant sows. Temperature was maintained at 22 ± 1 °C in the chambers and the relative humidity was controlled at 70 ± 5%.

Composition and nutritional content of all diets for Exp. 1 (Table 2) and Exp. 2 (Table 3) was adjusted according to the nutrient requirements of the NRC (2012) [10]. The concentrations of aflatoxin B1 and zearalenone in each corn DDGSs and diet (Table 4) were lower than the maximum thresholds specified for corn DDGSs by the GB/T 25866-2010 standard [15].

### 2.3. Sample Collection

Samples of corn DDGSs and test diets (50 g) were collected from the upper, middle and lower parts of each bag. These samples were then mixed thoroughly and sub-sampled via the quartering method. The sub-samples were then crushed and stored in sealed plastic bags at −4 °C until analysis.

In Exp. 1, from d 8 to d 12, about 100 g of fresh fecal samples (collected either from the anus or immediately after defecation, ensuring no contamination [16]) were gathered daily from each pig. The samples were placed in plastic bags and stored at −20 °C until analysis. At the end of Exp. 1, fecal samples from 8 sows per treatment group were thoroughly mixed, and about 300 g of subsamples were retained. These subsamples were oven-dried for 72 h at 65 °C, and subsequently allowed to equilateral with ambient moisture for about 24 h until a constant weight was achieved. The dried samples were then crushed and passed through a 40-mesh sieve, before being stored in sealed bags at −20 °C for chemical analysis.

In Exp. 2, during d 6–d 10, all feces from each pregnant sow were collected at 08:30 and stored at −20 °C. Urine was also collected at 08:30 for each pregnant sow, using plastic buckets containing 50 mL of 6 mol/L HCl. The collected urine was filtered through cotton gauze, and 10% of the daily urine volume was stored at −20 °C. At the end of Exp. 2, daily fecal samples were thawed and mixed, then a subsample (300 g) was stored for analysis. Daily urine samples were thawed and mixed and two subsamples (50 mL each) were retained for analysis. Fecal subsamples were oven-dried for 72 h at 65 °C, then were ground through a 40-mesh sieve. Urine was collected separately during the fasting period (d 6–d 10) for the calculation of FHP.

### 2.4. Chemical Analysis

Samples of corn DDGSs, diets and fecal were analyzed for DM (Method 934.01), Ash (Method 942.05) according to the procedures of the Association of Official Analytical Chemists (AOAC, 2012) [17], CP (CP = N × 6.25, procedure 976.05) in AOAC (2016) [18], EE [19], NDF and ADF [20]. Chromic content in diets and fecal samples was analyzed using the atomic absorption spectrophotometer (AOAC, 2006) [21]. The GE of corn DDGSs, diets, fecal and urine samples were determined using an isoperibol calorimeter (Parr 6400 Calorimeter, Moline, IL, USA). The OM concentration was calculated by the formula (OM = DM − Ash). Amino acids (AA) of SBM samples and diets were analyzed using an AA analyser (Hitachi L-8900; Hitachi Ltd., Tokyo, Japan) and high-performance liquid chromatography (Agilent 1200 Series; Agilent Technologies Inc., Santa Clara, CA, USA) according to GB/T 18246-2000 [22].

The contents of aflatoxin B_1_ and zearalenone were detected according to the instructions of the ELISA kit (Gold Standard Diagnostics, 1047 Budapest, Fóti út 56, Hungary).

### 2.5. Calculations

Exp. 1

The ATTD of dietary nutrients was calculated according to the following equation [23]:

ATTD of dietary nutrients (%) = (1 − (A/B) × (C/D)) × 100%(1)

Where A was the chromium content in the dietary samples, B represented the chromium content in fecal samples, C was the content of nutrients in fecal samples, and D was the content of the nutrient in the dietary samples.

The values of dietary DE and ME were calculated according to the following equations [24]:

DE of diets (MJ/kg) = Dietary energy digestibility (%) × GE of diet(2)

ME of diets (MJ/kg) = 0.997 × dietary DE − 0.68 × CP + 0.23 × EE(3)

The ATTD of nutrients in corn DDGSs was calculated according to the following equation [25]:

ATTD of corn DDGSs = (content of nutrient in the test diet) × ATTD of nutrient in the  test diet) − (1 − X%) × (nutrient content in basal diet × ATTD of nutrient in the basal  diet)/(X_1_% × content of nutrient in corn DDGSs) × 100 (4)

Where X% was the percentage of corn DDGSs to be tested in the test diet.

The DE and ME of corn DDGSs were calculated according to the following equations [21]:

DE (MJ/kg, DM) = [(DE of the test diet -DE of the basal diet × (1 − X_1_%)]/X_1_%/X_2_% (5)

ME (MJ/kg, DM) = [(ME of the test diet − ME of the basal diet × (1 − X_1_%)]/X_1_%/X_2_% (6)

Where X_1_% was the proportion of corn DDGSs replacing the energy components of the basal diet; X_2_% was the proportion of energy components (corn and soybean meal) in the basal diet.

Exp. 2

Total heat production (THP) and FHP of pigs were calculated for each day by gas exchange volumes and urinary N loss according to the following equation [26]:

THP or FHP (kJ) = 16.1753 × O_2_ (L) + 5.0208 × CO_2_ (L) − 2.1673 × CH_4_ (L) − 5.9873 × urinary N (g) (7)

The respiratory quotient (RQ) was calculated according to the following equation [27]:

RQ = CO_2_(L/d)/O_2_(L/d) (8)

Retained energy (RE), retained energy as protein (REP), and retained energy as lipid (REL) in various diets were calculated according to the following equations [27]:

RE (kJ) = ME intake (MJ) − THP(MJ)(9)

REP (kJ) = (nitrogen intake, g − fecal nitrogen, g − urine nitrogen, g) × 6.25 × 23.86 (kJ/g)(10)

REL (kJ) = net deposition energy (kJ) − protein deposition energy (kJ)(11)

The ATTD of diets and corn DDGSs was calculated according to the equations provided by Adeola et al. [28]:

ATTD of dietary nutrients (%) = (total amount of nutrient intake − total amount of  nutrient in feces)/total amount of nutrient intake(12)

ATTD of corn DDGSs nutrients (%) = (ATTD of a certain nutrient in the test diet −  (100% − X%) × ATTD of a certain nutrient in the basal diet(13)

Where X% represented the percentage of corn DDGSs in the test diet.

The DE [28], ME [28] and NE [27] concentration of diets were calculated according to the following equations:

DE (MJ/kg, DM) = (GE intake, MJ − fecal energy, MJ)/DM intake, kg(14)

ME (MJ/kg, DM) = (GE intake, MJ − fecal energy, MJ − urine energy, MJ − CH_4_ energy, MJ)/DM intake, kg (15)

NE (MJ/kg, DM) = (ME intake, MJ − THP, MJ + FHP, MJ)/DM, kg(16)

The DE, ME and NE of corn DDGSs were calculated according to the following equations [29]:

DE (MJ/kg, DM) = [DE of the test diet − DE of the basal diet × (1 − X_1_%)]/X_1_%/X_2_% (17)

ME (MJ/kg, DM) = [ME of the test diet − ME of the basal diet × (1 − X_1_%)]/X_1_%/X_2_% (18)

NE (MJ/kg, DM) = [NE of the test diet − NE of the basal diet) × (1 − X_1_%)]/X_1_%/X_2_% (19)

In these equations, X_1_% was the proportion of corn DDGSs replacing the energy components of the basal diet; X_2_% was the proportion of energy components (corn and soybean meal) in the basal diet.

### 2.6. Statistical Analysis

Data were analyzed using IBM SPSS Statistics (Version 24.0, IBM Corp., Armonk, NY, USA). In Exp. 1, we regarded different substitution levels of corn DDGSs as fixed effects, and pregnant sows as random effects. Linear and quadratic models were fitted to assess the effects of substitution levels on nutrient digestibility and available energy in corn DDGSs and the experimental diets. In Exp. 2, a mixed-effects model included dietary treatments as fixed effects, with experimental period, respiration chambers, replication, and sows within replication as random effects. A one-way ANOVA with Tukey’s HSD was utilized to determine significant difference (*p* < 0.05, *p* < 0.01). For predictive modeling, JMP Pro (v19.0, SAS Institute Inc., Cary, NC, USA) was used to analyze correlations between energy values and chemical composition. Stepwise regression was applied to develop prediction equations for DE, ME, and NE. The model fit was evaluated using R^2^, root mean square error (RMSE), and *p*-values.

## 3. Results

### 3.1. Chemical Composition of Diets in Exp. 1

Analyzed chemical composition (DM basis) of diets is presented in Table 5. Compared with basal-1, the contents of GE, DM, CP, NDF, ADF, EE, Ash and AAs in the test diets increased with the rising substitution level of corn DDGSs.

### 3.2. The ATTD of Nutrients and Available Energy in the Diets of Pregnant Sows in Exp. 1

As shown in Table 6 and Table 7, the ATTD of DM, OM, ADF, EE and GE in diets was affected both linearly and quadratically (*p* < 0.05) by the inclusion of corn DDGSs. As the substitution level of DDGSs increased from 20% to 50%, the ATTD of ADF decreased, and the ATTD of DM, OM, EE and GE peaked at the 30% substitution level. Meanwhile, the ATTD of NDF and DE, ME exhibited quadratic effects (*p* < 0.05) in diets and reached the maximum at the 30% substitution level. The CV of DE and ME in D30 decreased by 2.0%, 2.6%, 0.3%, 0.8%, and 0.3%, 2.2%, 0.9%, 1.4%, compared to D20, D40, and D50, respectively.

### 3.3. The ATTD and CV of Nutrients in the Corn DDGSs of Pregnant Sows in Exp. 1

As illustrated in Table 8, with the increasing substitution level of corn DDGSs increased, the ATTD of ADF decreased linearly or quadratically (*p* < 0.05), while the ATTD of CP increased linearly or quadratically (*p* < 0.05). A significant quadratic effect was observed for the ATTD of EE, DE, and ME in corn DDGSs (*p* < 0.05). The highest DE and ME values and the lowest CV in DE and ME of corn DDGSs were observed at a substitution level of 30% (Table 9).

### 3.4. Chemical Composition of Diets in Exp. 2

The nutritional composition of corn DDGSs and diets is presented in Table 10 and Table 11, respectively. Compared with basic diet, the test diets supplemented with 30% corn DDGSs exhibited a numerical increase in GE, CP, NDF, ADF, EE and Ash content. The CV of GE, EE, NDF, ADF and Ash content in the five sources of corn DDGSs were below 10%, while the CV of EE was above 30% (35.8%) (Table 11).

### 3.5. ATTD of Nutrients and Nitrogen Balance in Diets in Exp. 2

As shown in Table 12, significant differences in nutrient digestibility were observed among different diets. Compared with other test diets, the ATTD of DM, OM, and GE in J2-D2 and X4-D4 was significantly decreased (*p* < 0.01). The ATTD of CP was significantly higher in J1-D1, N3-D3 and C5-D5 (*p* < 0.01) than that in X4-D4. Conversely, the ATTD of NDF was lower in J2-D2 and X4-D4 (*p* < 0.01), compared to the N3-D3 and C5-D5. The ATTD of ADF was higher in J1-D1 and N3-D3 (*p* < 0.01) than in X4-D4 and C5-D5 diets. The ATTD of EE in the J2-D2 and X4-D4 was significantly decreased (*p* < 0.01) compared to the N3-D3 and C5-D5. Additionally, nitrogen intake of pregnant sows in the test diets was significantly higher than that in the basal-2 (*p* < 0.01). There were no significant differences in fecal nitrogen, urine nitrogen, or nitrogen retention among the test diets.

As indicated in Table 13, no significant differences in energy balance, respiratory quotient, or concentrations of DE, ME, NE, or energy utilization were observed among different diets. However, an unexpected decrease in body weight and a negative energy retention as lipid (RE_L_) were noted in all groups of pregnant sows at the end of Exp. 2.

### 3.6. ATTD of Nutrients and Available Energy Values of Different Corn DDGSs Samples in Exp. 2

As shown in Table 14, the type of corn DDGS had a significant effect on the ATTD of DM, GE, NDF, ADF, EE, and OM (*p* < 0.01). The ATTD of OM and GE in J1, N3 and C5 was higher than that in J2 and X4 (*p* < 0.01). The ATTD of NDF in C5 was significantly higher than that in J2 and X4 (*p* < 0.01), and the ATTD of EE in N3 and C5 was significantly higher than that of J2 and X4 (*p* < 0.01). The NE of J1 was significantly lower than that of N3, and C5 (*p* < 0.05). There was a trend of differences in ME among different corn DDGS samples, but no differences were observed in DE. The concentrations of DE, ME and NE ranged from 15.58 to 18.07 MJ/kg DM, 12.17 to 16.42 MJ/kg DM, and 8.76 to 15.88 MJ/kg DM, respectively. No significant differences were observed in the ratios of ME/DE and NE/ME.

### 3.7. Correlation Between Available Energy Value and Chemical Composition, and the Prediction Equation for Corn DDGSs in Exp. 2

As shown in Table 15, the CP was significantly positively correlated with DE (*p* < 0.05). EE was significantly positively correlated with NE (*p* < 0.05). NDF and Hemicellulose (HC) were negatively correlated with ME (*p* < 0.05). The predictive equations (Table 16) for DE, ME, and NE of corn DDGSs were: DE = −0.86 + 0.58 CP + 0.12 EE (R^2^ = 0.98, RMSE = 0.16, *p*= 0.01), ME = 27.07 + 0.19 EE − 0.61 HC (R^2^ = 0.84, RMSE = 0.78, *p* = 0.08), NE = 23.45 − 0.45 NDF + 0.47 EE (R^2^ = 0.98, RMSE = 0.30, *p* = 0.01). In Table 17, the highest absolute value of relative error (|RE|) observed between the predicted and determined values was 5.81%, while other values were below 5%. The |RE| between the predicted and measured values of DE is lower than that of ME and NE.

## 4. Discussion

### 4.1. ATTD, Available Energy and CV of Dietary and Corn DDGSs in Exp. 1

Among the test groups, the 30% substitution level exhibited the highest ATTD of dietary nutrients. The nutrient content of the test diets increased through the use of corn DDGSs to replace the energy-supplying components of the basal diet, especially fiber content. Insoluble fiber which constitutes the majority of the fiber in corn DDGSs is poorly digested and fermented in the gastrointestinal tract of pigs [30,31,32]. Therefore, increasing the insoluble fiber level in the form of DDGSs decreased the digestibility of most dietary components [33]. Xu [1] reported that the ATTD of DM, CP, and GE in diets of pregnant sows decreased linearly with increasing supplemental levels of corn DDGSs. A negative correlation between dietary fiber (DF) and digestibility of nutrients from other ingredients is well established [33]. However, the digestibility of EE does not generally follow the same pattern, as demonstrated in other studies [34]. As substitution level of corn DDGSs increased, the dietary EE content also increased. With the increase in feed intake, the ATTD of EE also increased [35,36,37], indicating that the endogenous amount of fat exerts a stronger influence on the ATTD of fat at low dietary levels than at higher levels, and the dietary fat from the diet has a lower digestibility than that from the fat source [35].

When the substitution level of corn DDGSs ranged from 20% to 50%, the ATTD of ADF in corn DDGSs decreased linearly or quadratically. The 30% substitution level exhibited the highest ATTD of DM, OM, EE, and GE in corn DDGSs. This observation can be explained by the fact that as the substitution level of corn DDGSs increased, the fiber content surpassed the tolerance of pregnant sows [38,39]. Furthermore, the high fiber content likely accelerated passage rate of chyme through the intestine, which in turn hindered the contact between digestive enzymes and chyme, ultimately leading to a reduction in the ATTD of nutrients [40]. It was found that the ATTD of dietary EE was the highest when the supplementation of corn DDGSs was at 45% [1]. The differences observed in comparison with Exp. 1 may be attributed to variations in the feed formula. Corn soybean basal diet was supplemented with corn oil in Xu’s experiment, which likely resulted in different fat compositions and contents. Research has confirmed that digestibility of added oil in the diet is higher than that of oil naturally contained in corn DDGSs [41,42].

Consistent with the ATTD of EE mentioned earlier, the ATTD of dietary CP typically does not follow the same pattern of influence from dietary fiber on ATTD [34], instead, the dietary CP content itself has been shown to have a positive effect on ATTD of CP [43]. Compared with basal diet, there was no significant difference in ATTD of CP in the test diets. However, the increase in substitution level of corn DDGSs led to a linear or quadratic increase in ATTD of CP in corn DDGSs. The results may be related to nitrogen (N) metabolism, including factors such as N intake and excretion. The ATTD of CP is calculated by taking the difference between CP intake and fecal CP excretion, and then dividing that by CP intake. Fecal CP includes both undigested CP excretion from diet and the endogenous losses of CP. As CP intake increases, the proportion of basal endogenous losses of CP to total CP intake decreases, resulting in an increase in the ATTD of CP [29], which aligns with the finding of Xue et al. [29]. Consequently, in future studies, it is advisable to focus on the interactions between fiber, CP and oil, as these factors may affect the dynamics of nutrient absorption [44].

Among all dietary groups, the DE and ME contents were highest in the 30% corn DDGSs. This may be related to the ATTD of GE. When corn DDGSs substitution exceeded 30%, there was a linear decrease in the ATTD of GE in the diet of pregnant sows, leading to a reduction in the available energy value. The DE and ME of corn DDGSs in the 30% substitution group were closest to those specified in Nutrient Requirement of Swine in China [40]. Additionally, the CV for DE and ME was smallest at the 30% substitution level. The substitution level of an ingredient can significantly influence the CV of energy values among different treatments [45]. In theory, as the substitution level of an ingredient increased, the measured available energy value is expected to be closer to the true value [8]. However, excessive substitution can create nutrient disparities between the basal and the test diets, which can negatively impact the evaluation of the available energy of tested ingredient [46]. Therefore, the minimum CV observed at the 30% substitution level indicates that the data measured at this level exhibited greater stability and repeatability [47]. This finding implies that the 30% substitution level is optimal for obtaining reliable and consistent energy values measurements for corn DDGSs in pregnant sows using the substitution method.

### 4.2. Nutritional Composition and Available Energy of Five Sources of Corn DDGSs in Exp. 2

Corn DDGS samples categorized based on their oil content: DDGSs 1 (J1) was classified as low oil, DDGSs 4 (X4) as medium oil, corn DDGSs 2, 3, and 5 (J2, N3 and C5) as high oil. The average EE content for these three categories was 3.92%, 9.98% and 11.87% (DM basis), respectively. These values are consistent with the contents provided by NRC (2012) [10]. Furthermore, the average values for NDF and ADF across the five corn DDGS samples were 35.01% and 10.43%, respectively. These values are comparable to those reported by Rech (NDF 36.84%; ADF 12.95%) [48]. The average CP content was 25.51%, which falls within the range of CP content (28.2% to 31.48%) observed in 107 corn DDGS samples retrieved by Rech [48]. The CV for EE was the highest among all nutritional components, while the CVs for other nutrients were below 10%. This indicates that the oil extraction process primarily affected the EE content and had minimal impact on other nutrients [1]. These findings are in line with previous observations and highlight the importance of considering oil content when evaluating the nutritional value of corn DDGSs.

The nutritional compositions of the test diets were influenced by the type of corn DDGSs used, particularly due to the high NDF content in J2 and X4, which subsequently resulted in high fiber content in test diet 2 (J2-D2) and 4 (X4-D4). The ATTD of DM, OM and GE in J2-D2, X4-D4 and J2, X4 significantly decreased. Meanwhile, the ATTD of EE in J2-D2 and the ATTD of NDF in X4 are the lowest. High levels of dietary fiber could decrease the digestibility of energy and nutrients in sows [49,50]. It should be noted that most of the fiber present in DDGSs is insoluble, for which the main fermentation site is the colon [50]. Insoluble dietary fiber can reduce the exposure time of digesta to enzymes and hindgut microbiota, increase the passage rate of the nutrient flow, and have a significant influence on the large intestinal turnover [34]. Therefore, with the inclusion of DDGSs, greater insoluble fiber levels in the form of DDGSs conducted the decreased digestibility of dietary components [32]. Despite a certain increase in fiber content of N3-D3 and C5-D5, their elevated oil content can partially mitigate the detrimental effects associated with high fiber [51], thereby slowing down gastric emptying and enhancing the ATTD of nutrients [52]. Therefore, test groups 3 and 5 had higher ATTD of dietary CP and NDF and higher ATTD of EE in corn DDGSs. These findings were consistent with results [1] that high fat content in corn DDGSs increased the ATTD of CP in the diet of pregnant sows. Furthermore, an elevated N intake can also affect the ATTD of CP [53,54]. These finding suggests that the digestibility of dietary fiber is not solely determined by the level of dietary fiber [45]. Instead, other factors such as the interaction between fiber, EE and CP in diets can also play a significant role in affecting the digestibility of nutrients [55].

The low N intake of pregnant sows in the control group was attributed primarily to the low CP content of the basic diet which is similar to the findings of Wang [56]. Compared with pigs fed the basal diet, pigs fed corn DDGSs diets had greater urinary N output, which indicates that some of the amino acids supplied by corn DDGSs were deaminated and used for energetic purposes [57]. Despite a decrease in the ATTD of CP in J2-D2, C5-D5 and basal diet-2, N retention remained unaffected. In fact, when the diet adequately supplies amino acids which may not compromise the N retention in pigs [58]. The results indicate that neither the total heat production (THP) nor fasting heat production (FHP) was significantly affected by different diets including 30% corn DDGSs. This finding is consistent with results from other studies, which also reported that THP is not affected by fiber level [56,59]. The reason for this lack of effect on THP and FHP may be attributed to the lower fiber content (15.85–25.8%) of the diets. In contrast, previous studies that observed an impact of fiber on the heat production (HP) typically used diets with high fiber levels (30–40%) [60]. Schrama [61] suggested that the HP related to physical activity decreases when fiber is increased in diets fed to growing pigs, leading to an overall reduction in HP. This implies that the physical activity of the pigs may be a contributing factor to the HP observed.

Ramonet et al. [62] discovered a positive correlation between the length of gestational days and calorie production during a fasting period. Their findings indicated that as gestation advances, calorie production increases. Pregnant sows used in our study were in mid-gestation (approximately 50 days), which is a later stage compared to the 30 days of gestation in the sows used by Wang [56]. This longer gestation period could be a factor in the relatively higher average calorie production observed during fasting in the pregnant sows of Exp. 2. Although we supplied 1.3 times the required amount of ME for maintain metabolism as recommended by the NRC (2012) [10]; the efficiency of ME utilization (RE_L_) was negative. This negative retention indicated that when sows were fed diets at this level of ME for maintain metabolism, sows tended to catabolize fat for energy to meet the energy demands of protein deposition. This conclusion aligned with Quiniou’s result which showed that pigs depend on body fat catabolism for protein deposition when feed intake is at or below maintenance levels [63]. Furthermore, the RQ of each group was less than 1, and the body weight of the pregnant sows decreased over the course of the experiment. These observations collectively confirm that the sows were in a state of catabolism. Similarly, Li observed that when the ME intake was below 1.4 times ME, the RQ of the sows was below 1.0, indicating an imbalance in energy status [64]. The above phenomenon reveals that when an animal’s energy intake is restricted, whether due to dietary design factors or increased metabolic requirements, body fat is preferentially used as an oxidative energy substrate to ensure the distribution of nutrients such as glucose and protein to vital organs [65]. Therefore, it may be necessary to increase energy intake levels to regulate this imbalance and ensure the nutritional needs of the pregnant sows are met in future study.

### 4.3. Correlation and Prediction Equations for Available Energy of Corn DDGSs for Pregnant Sows

Energy is the most expensive component of diets. Therefore, accurately determining energy content of feed ingredients is important [66]. In Exp. 2, the |RE| ranges for DE, ME, and NE in Experiment 2, NRC (2012) [10] and Nutrient requirements for pigs [40] were 3.3–16.5%, 11.48–31.11%, 2.65–14.14%, respectively. It is evident that the energy content of feed ingredients is not fixed. Factors such as the origin of the ingredients [67] and the specific type or length of the fermentation process [66] can potentially lead to substantial variations in their nutritional profile. Furthermore, the growth stages of animals can also influence the available energy of these ingredients [7]. Therefore, relying on the recommended values from NRC (2012) [10] or Nutrient requirements for pigs [40], or other static compositions can be risky, as it may lead to inaccurate feed formulations due to the wide range of variability in the nutritional composition and digestibility of feed ingredients. An effective solution to these challenges is the establishment of dynamic models that utilize conventional chemical compositions to predict the available energy in feed ingredients [24]. The closer the determined values align with the predicted values, the more reliable the equations become. Kim et al. [68] demonstrated that excessively high dietary CP increases urinary energy loss, leading to an underestimation of the ME of test ingredient. They recommended keeping dietary CP less than 170 g/kg (as-fed basis) to accurately determine the available energy of soy protein sources. In Exp. 1, substituting 30% of the energy components in the basal diet with corn DDGSs produced in a dietary CP content of 14.71% (DM)-well within the guideline proposed by Kim. This alignment further ensured the reliability of the ME data obtained in Exp. 2. In Exp. 2, the test diets contained 16.44–17.79% CP (DM basis), equivalent to 10.96–16.80% CP as-fed basis, again remaining below the 17% (170 g/kg) threshold. These results confirm that the 30% corn DDGSs inclusion rate chosen in Exp. 1 was appropriate and the ME values obtained in Exp. 2 are reliable.

The DE and ME contents were negatively correlated with the fiber components. EE showed strong positive correlation with NE contents in pregnant sows which was consistent with previous reports [6,69,70,71]. CP was significantly positively correlated with DE. DE prediction equations of 25 corn DDGS samples established by Li et al. [6] also included CP as a positively correlated predictive factor, DE and ME values in corn DDGSs were related to EE and fiber concentrations. Kerr et al. [4] indicated that measures of dietary fiber, such as ADF or TDF, were important in determining the DE or ME content of corn DDGSs for growing pigs. Similarly, EE and the fiber content were also the main predictors in the prediction equation established for this experiment. The *p* value for ME prediction equation did not reach the conventional statistical significance level (*p* < 0.05), most likely because the sample size of corn DDGSs was still limited. Consequently, we will enlarge this sample in future work to refine the equation. At the same time, model reliability should not be judged by the *p* value alone, the equation exhibited R^2^ > 0.8, and the relative error between the predicted values and the observed values was only 6%-both indicators confirming a robust fit.

The chemical composition of the five tested corn DDGS samples with big difference in EE was input into the prediction equations to obtain the predicted values. Notably, the relative error between the determined and predicted values of ME for N3 was the only one that surpassed the 5%. The DE range of corn DDGS samples fell within the ranges provided by NRC (2012) [10] and the Nutrient requirements for pigs [40]; however, the ME and NE of low-oil corn DDGSs were below the ranges of both, while those of high-oil corn DDGSs exceed the ranges of both. This might be related to the processing technology of corn DDGSs. Studies have shown that feed intake levels in growing pigs have no significant effect on DE and ME of corn–soybean meal-based diets, as well as the ratio of DE to ME [72,73]. Therefore, the available energy values measured at 1.3 times the required amount of ME for maintain metabolism exhibited a certain degree of reliability. Above conclusion was only a general conclusion, specifically, whether the feeding level of pregnant sows had any impact on the determination of available energy values still required further experimental verification. Meanwhile, the range of corn DDGS sources must be expanded, more representative predictive equations for DE, ME, and NE of corn DDGSs in pregnant sows can be derived and subsequently validated through animal experiments.

## 5. Conclusions

The proper level of corn DDGSs as a substitute for energy-supplying components in the basal diet of pregnant sows is 30%. The chemical characteristics of corn DDGSs, particularly the content of EE, are influenced by processing methods, leading to a range of available energy values. Correlation analysis revealed that EE and fiber components are the primary predictors of DE, ME, and NE values in corn DDGSs for pregnant sows. By establishing dynamic prediction equations for corn DDGSs, it is possible to achieve precise formulation and enhance feed utilization.

## Figures and Tables

**Table 1:** Nutrient content of corn DDGSs for Exp. 1 and Exp. 2 (%, DM basis).

Item ^1^	Corn DDGSs					
	Exp. 1	Exp. 2 ^2^				
		J1	J2	N3	X4	C5
GE (MJ/kg)	21.46	19.56	20.33	22.23	20.12	22.15
CP	27.83	28.42	28.54	29.99	26.23	29.36
NDF	30.91	36.68	38.19	34.41	35.9	29.88
ADF	13.42	10.72	11.00	10.38	10.97	9.09
EE	12.40	3.92	11.47	12.00	9.98	12.15
Ash	4.20	5.16	5.27	5.39	5.02	5.00
Indispensable AA						
Arg	1.11	1.27	1.08	1.19	1.05	1.06
His	0.71	0.85	0.70	0.75	0.71	0.71
Leu	3.41	3.88	3.12	3.33	3.03	3.49
Ile	1.02	1.14	0.93	0.98	0.92	1.02
Lys	0.71	0.97	0.84	0.88	0.80	0.74
Met	0.55	0.69	0.6	0.61	0.50	0.58
Phe	1.33	1.59	1.29	1.39	1.19	1.39
Thr	1.11	1.2	1.01	1.04	0.98	1.05
Trp	0.17	0.18	0.19	0.19	0.16	0.21
Val	1.37	1.55	1.28	1.36	1.23	1.33
Dispensable AA						
Ala	2.18	2.31	1.93	2.09	1.92	2.07
Asp	1.87	1.97	1.7	1.7	1.58	1.7
Cys	0.5	0.59	0.52	0.51	0.48	0.5
Glu	5.39	5.54	4.81	4.94	4.52	5.05
Gly	1.09	1.26	1.09	1.17	1.04	1.07
Pro	2.47	2.71	2.19	2.4	2.19	2.49
Ser	1.38	1.52	1.26	1.32	1.22	1.34
Tyr	0.97	1.12	1.01	1.16	0.95	1.16

^1^ GE, gross energy; CP, crude protein; NDF, neutral detergent fiber; ADF, acid detergent fiber; EE, ether extract; Ash, crude ash; AA, amino acid; Arg, arginine; His, histidine; Leu, leucine; Ile, Isoleucine; Lys, Lysine; Met, methionine; Phe, phenylalanine; Thr, threonine; Trp, tryptophan; Val, valine; Ala, alanine; Asp, aspartic; Cys, cystine; Gly, glycine; Pro, proline; Ser, serine; Tyr, tyrosine. ^2^ J1: Heilongjiang Shenglong Industrial Co., Ltd. (Harbin, China); J2: Hei-longjiang Zhongke Green Biotechnology Co., Ltd. (Mudanjiang, China); N3: Henan Mengzhou Huaxing Co., Ltd. (Jiaozuo, China); X4: Xinjiang Bosheng Liquor Brewing Co., Ltd. (Bortala Mongolian Autonomous Prefecture, China); C5: Chifeng Ruiyang Chemical Co., Ltd. (Chifeng, China).

**Table 2:** Composition and nutritional content of the diets in Exp. 1 (%, as fed basis).

Item	Basal-1 ^1^	Test Diets ^2^			
		D20	D30	D40	D50
Corn	90.00	72.09	63.19	54.00	44.81
Soybean meal	6.70	5.37	4.70	4.02	3.34
Corn DDGSs	0.00	19.34	29.01	38.68	48.35
Limestone	0.80	1.10	1.20	1.40	1.50
Dicalcium phosphate	1.30	0.90	0.70	0.70	0.80
Salt	0.40	0.40	0.40	0.40	0.40
Premix ^3^	0.50	0.50	0.50	0.50	0.50
Chromic oxide	0.30	0.30	0.30	0.30	0.30
Total	100	100	100	100	100
Calculated nutrient levels ^4^					
ME (MJ/kg)	14.46	14.52	14.57	14.56	14.55
NE (MJ/kg)	11.35	11.18	11.1	10.98	10.87
CP	10.10	13.60	15.33	17.03	18.73
Ca	0.63	0.65	0.65	0.72	0.78
P	0.48	0.49	0.50	0.54	0.60
Lys	0.33	0.37	0.38	0.40	0.42
Met	0.18	0.23	0.25	0.27	0.30
Thr	0.28	0.37	0.41	0.46	0.50
Trp	0.07	0.07	0.07	0.08	0.08

^1^ Basal-1, corn–soybean basal diet. ^2^ D20–D50 were test diets which replaced energy supplements in the basic diet with corn DDGSs at levels of 20%, 30%, 40% and 50%, respectively. ^3^ Premix provided per kilogram of complete feed: vitamin A, 11,000 IU; vitamin D_3_, 3000 IU; vitamin E, 15.0 IU; vitaminK_3_, 1.6 mg; vitamin B_1_, 1.5 mg; vitamin B_2_, 3.0 mg; vitamin B_4_, 1.5 mg; vitamin B_12_, 0.02 mg; nicotinamide, 22.5 mg; pantothenic acid, 15.0 mg; folic acid, 2.5 mg; biotin, 0.2 mg; iron, 400.0 mg; copper, 16.5 mg; zinc, 75.0 mg; manganese, 35.0 mg; iodine, 1.0 mg; and selenium, 0.3 mg. ^4^ Nutrient levels were based on the values provided by the NRC (2012) [10]. ME; metabolizable energy; NE, net energy; CP, crude protein; Ca, calcium; P, phosphorus; Lys, Lysine; Met, methionine; Thr, threonine; and Trp, tryptophan; The concentrations of Lys, Met, Thr, and Trp were determined as total amino acids.

**Table 3:** Composition and nutritional content of the diets in Exp. 2 (%, as fed basis).

Item	Basal-2 ^1^	Test Diets ^2^				
		J1-D1	J2-D2	N3-D3	X4-D4	C5-D5
Corn	90.00	63.19	63.19	63.19	63.19	63.19
Soybean meal	7.00	4.91	4.91	4.91	4.91	4.91
Corn DDGSs J1	-	29.10	-	-	-	-
Corn DDGSs J2	-	-	29.10	-	-	-
Corn DDGSs N3	-	-	-	29.10	-	-
Corn DDGSs X4	-	-	-	-	29.10	-
Corn DDGSs C5	-	-	-	-	-	29.10
Limestone	0.80	1.20	1.20	1.20	1.20	1.20
Dicalcium phosphate	1.30	0.70	0.70	0.70	0.70	0.70
Salt	0.40	0.40	0.40	0.40	0.40	0.40
Premix ^3^	0.50	0.50	0.50	0.50	0.50	0.50
Total	100.00	100.00	100.00	100.00	100.00	100.00
Calculated nutrient levels ^4^						
ME (MJ/kg)	14.50	14.61	14.61	14.61	14.61	14.61
NE (MJ/kg)	11.38	11.13	11.13	11.13	11.13	11.13
CP	11.41	17.79	16.52	17.68	16.44	16.60
Ca	0.63	0.65	0.65	0.65	0.65	0.65
P	0.48	0.50	0.50	0.50	0.50	0.50
Lys	0.45	0.65	0.62	0.63	0.54	0.52
Met	0.23	0.37	0.30	0.36	0.29	0.34
Thr	0.29	0.71	0.62	0.58	0.53	0.53
Trp	0.13	0.14	0.11	0.13	0.15	0.10

^1^ Basal-2, corn–soybean basal diet. ^2^ J1: Heilongjiang Shenglong Industrial Co., Ltd. (Harbin, China); J2: Hei-longjiang Zhongke Green Biotechnology Co., Ltd. (Mudanjiang, China); N3: Henan Mengzhou Huaxing Co., Ltd. (Jiaozuo, China); X4: Xinjiang Bosheng Liquor Brewing Co., Ltd. (Bortala Mongolian Autonomous Prefecture, China); C5: Chifeng Ruiyang Chemical Co., Ltd. (Chifeng, China). J1-D1, J2-D2, N3-D3, X4-D4 and C5-D5 were test diets containing 30% corn DDGSs from 5 different sources. ^2^ Premix provided is same as Exp. 1. ^3^ Premix provided per kilogram of complete feed: vitamin A, 11,000 IU; vitamin D_3_, 3000 IU; vitamin E, 15.0 IU; vitaminK_3_, 1.6 mg; vitamin B_1_, 1.5 mg; vitamin B_2_, 3.0 mg; vitamin B_4_, 1.5 mg; vitamin B_12_, 0.02 mg; nicotinamide, 22.5 mg; pantothenic acid, 15.0 mg; folic acid, 2.5 mg; biotin, 0.2 mg; iron, 400.0 mg; copper, 16.5 mg; zinc, 75.0 mg; manganese, 35.0 mg; iodine, 1.0 mg; and selenium, 0.3 mg. ^4^ Nutrient levels based on the raw material energy values provided according to the NRC (2012) [10]. ME; metabolizable energy; NE, net energy; CP, crude protein; Ca, calcium; P, phosphorus; Lys, Lysine; Met, methionine; Thr, threonine; Trp, tryptophan. The concentrations of Lys, Met, Thr, and Trp were determined as total amino acids.

**Table 4:** Mycotoxin content in corn DDGSs and diets (μg/kg, as fed basis).

Exp	Item	Aflatoxin B_1_	Zearalenone
Exp. 1 ^1^	Corn DDGSs	<1	416.62
	Basal diet	1.88	87.93
	D20	<1	145.55
	D30	<1	240.94
	D40	<1	301.63
	D50	<1	351.38
Exp. 2 ^2^	Corn DDGSs J1	<1	388.64
	Corn DDGSs J2	<1	268.81
	Corn DDGSs N3	<1	466.79
	Corn DDGSs X4	<1	60.12
	Corn DDGSs C5	<1	437.09
	Basal diet	<1	350.37
	J1-D1	<1	250.14
	J2-D2	<1	226.80
	N3-D3	<1	463.71
	X4-D4	<1	159.15
	C5-D5	<1	220.36

^1^ Basal-1, corn–soybean basal diet; D20–D50 were test diets which replaced energy supplements in the basic diet with corn DDGSs at levels of 20%, 30%, 40% and 50%, respectively. ^2^ Premix provided per kilogram of complete feed: vitamin A, 11,000 IU; vitamin D_3_, 3000 IU; vitamin E, 15.0 IU; vitaminK_3_, 1.6 mg; vitamin B_1_, 1.5 mg; vitamin B_2_, 3.0 mg; vitamin B_4_, 1.5 mg; vitamin B_12_, 0.02 mg; nicotinamide, 22.5 mg; pantothenic acid, 15.0 mg; folic acid, 2.5 mg; biotin, 0.2 mg; iron, 400.0 mg; copper, 16.5 mg; zinc, 75.0 mg; manganese, 35.0 mg; iodine, 1.0 mg; selenium, 0.3 mg. Basal-2, corn–soybean basal diet; J1-D1, J2-D2, N3-D3, X4-D4 and C5-D5 were test diets containing 30% corn DDGSs from 5 different sources.

**Table 5:** Chemical composition of the different diets (%, DM basis).

Item	Basal-1 ^1^	Test Diets			
		D20	D30	D40	D50
GE (MJ/kg)	16.01	16.63	17.13	17.46	17.68
DM	88.79	89.74	89.88	90.30	90.65
CP	10.50	13.30	14.71	16.67	18.48
NDF	13.00	17.16	18.28	20.12	21.42
ADF	4.25	5.83	6.19	6.92	7.13
EE	2.89	4.49	6.22	6.72	7.36
Ash	3.96	4.43	4.71	5.28	5.79
Indispensable AA					
Arg	0.50	0.61	0.72	0.74	0.83
His	0.26	0.35	0.44	0.47	0.56
Leu	1.00	1.41	1.86	1.98	2.45
Ile	0.33	0.42	0.57	0.60	0.73
Lys	0.42	0.43	0.54	0.56	0.63
Met	0.22	0.26	0.30	0.37	0.40
Phe	0.57	0.66	0.79	0.89	0.98
Thr	0.33	0.46	0.62	0.62	0.76
Trp	0.08	0.12	0.13	0.16	0.12
Val	0.46	0.59	0.77	0.81	0.96
Dispensable AA					
Ala	0.63	0.89	1.14	1.21	1.47
Asp	0.65	0.82	1.13	1.06	1.35
Cys	0.21	0.25	0.28	0.31	0.35
Glu	1.66	2.21	2.95	3.00	3.73
Gly	0.38	0.50	0.63	0.67	0.77
Pro	0.85	1.00	1.30	1.35	1.64
Ser	0.44	0.59	0.78	0.79	0.98
Tyr	0.31	0.50	0.54	0.63	0.69

^1^ Basal-1, corn–soybean basal diet; D20–D50 were test diets which replaced energy supplement in the basic diet with corn DDGSs at levels of 20%, 30%, 40% and 50%, respectively. GE, gross energy; DM, dry matter; CP, crude protein; NDF, neutral detergent fiber; ADF, acid detergent fiber; EE, ether extract; Ash, crude Ash; AA, amino acid; Arg, arginine; His, histidine; Leu, leucine; Ile, Isoleucine; Lys, Lysine; Met, methionine; Phe, phenylalanine; Thr, threonine; Trp, tryptophan; Val, valine; Ala, alanine; Asp, aspartic; Cys, cystine; Gly, glycine; Pro, proline; Ser, serine; Tyr, tyrosine.

**Table 6:** Effects of different corn DDGSs substitution levels on ATTD of nutrients and the available energy in diet of pregnant sows (%).

Item	Basal-1	Test Diets				SEM	*p* Value	
		D20	D30	D40	D50		Linear	Quadratic
DM	87.92	84.47	85.47	79.75	77.65	0.36	<0.01	<0.01
OM	90.73	87.87	88.23	83.01	80.48	0.32	<0.01	<0.01
NDF	69.82	64.47	88.73	58.06	55.65	0.95	0.10	<0.01
ADF	68.64	67.01	66.70	63.68	61.62	0.73	<0.01	<0.01
EE	58.22	61.46	71.67	66.71	66.03	0.78	0.03	<0.01
CP	82.83	81.86	82.86	80.94	81.12	0.32	0.12	0.26
GE	87.93	84.88	86.08	80.73	78.65	0.35	<0.01	<0.01
DE (MJ/kg)	14.08	14.12	14.74	14.09	13.81	0.06	0.11	<0.01
ME (MJ/kg)	13.97	13.99	14.61	13.95	13.66	0.06	0.07	<0.01

Basal-1, Corn–soybean basal diet; D20–D50 were test diets which replaced energy supplements in the basic diet with corn DDGSs at levels of 20%, 30%, 40% and 50%, respectively. DM, dry matter; OM, organic matter; NDF, neutral detergent fiber; ADF, acid detergent fiber; EE, ether extract; CP, crude protein; GE, gross energy; DE, digestible energy; ME; metabolizable energy; SEM, standard error of the mean.

**Table 7:** Effect of different substitution levels on the CV of available energy values in diets for pregnant sows (%).

Item	Basal-1	Test Diets			
		D20	D30	D40	D50
CV of DE in diets	3.7	3.3	1.7	2.0	2.5
CV of ME in diets	1.4	3.3	1.1	2.0	2.5

CV, coefficients of variation; DE, digestible energy; ME; metabolizable energy. Basal-1, corn–soybean basal diet; D20–D50 were test diets which replaced energy supplements in the basic diet with corn DDGSs at levels of 20%, 30%, 40% and 50%, respectively.

**Table 8:** Effects of different substitution levels on ATTD of DDGSs nutrients in pregnant sows (%).

Item	Test Diets				SEM	*p* Value	
	D20	D30	D40	D50		Linear	Quadratic
DM	72.99	81.15	68.66	67.17	1.40	0.70	0.59
OM	76.79	81.53	69.70	68.10	1.21	0.24	0.39
ADF	63.36	63.15	54.79	45.97	1.65	0.02	0.04
EE	64.59	91.43	79.20	73.25	1.55	0.38	<0.01
CP	72.87	81.06	82.82	85.44	0.83	<0.01	<0.01
GE	76.00	85.16	72.38	69.29	1.47	0.43	0.39
DE (MJ/kg)	14.06	16.33	14.33	13.82	1.26	0.36	0.03
ME (MJ/kg)	13.87	16.14	14.14	13.62	1.26	0.36	0.03

D20–D50 were test diets which replaced energy supplements in the basic diet with corn DDGSs at levels of 20%, 30%, 40% and 50%, respectively. DM, dry matter; OM, organic matter; ADF, acid detergent fiber; EE, ether extract; CP, crude protein; GE, gross energy; DE, digestible energy; ME; metabolizable energy; SEM, standard error of the mean.

**Table 9:** Effect of different substitution levels on the CV of available energy in corn DDGSs (%).

Item	Test Diets			
	D20	D30	D40	D50
CV of DE in corn DDGSs	14.94	4.62	9.73	9.43
CV of ME in corn DDGSs	15.10	4.66	9.90	9.65

D20–D50 were test diets which replaced energy supplements in the basic diet with corn DDGSs at levels of 20%, 30%, 40% and 50%, respectively. CV, coefficients of variation; DE, digestible energy; ME; metabolizable energy.

**Table 10:** Nutrient content of basal and test diets (%, DM basis).

Item ^3^	Basal-2 ^1^	Test Diets ^2^				
		J1-D1	J2-D2	N3-D3	X4-D4	C5-D5
GE (MJ/kg)	18.17	18.35	18.65	18.64	18.30	18.59
CP	11.41	17.79	16.52	17.68	16.44	16.60
NDF	12.16	19.43	19.44	18.86	19.84	17.62
ADF	3.69	5.54	6.36	6.01	4.60	4.26
EE	3.28	3.29	5.94	4.84	5.37	5.71
Ash	3.97	4.96	5.20	4.99	4.89	4.80
Indispensable AA						
Arg	0.51	0.80	0.76	0.77	0.72	0.67
His	0.30	0.49	0.44	0.42	0.39	0.40
Leu	1.35	2.18	1.87	1.82	1.69	1.77
Ile	0.46	0.73	0.61	0.58	0.51	0.53
Lys	0.45	0.65	0.62	0.63	0.54	0.52
Met	0.23	0.37	0.30	0.36	0.29	0.34
Phe	0.60	0.95	0.78	0.79	0.73	0.73
Thr	0.29	0.71	0.62	0.58	0.53	0.53
Trp	0.13	0.14	0.11	0.13	0.15	0.10
Val	0.50	0.86	0.77	0.75	0.69	0.71
Dispensable AA						
Ala	0.72	1.25	1.13	1.13	1.06	1.09
Asp	0.88	1.35	1.20	1.10	0.98	0.99
Cys	0.20	0.31	0.31	0.29	0.27	0.29
Glu	2.10	3.36	2.99	2.82	2.61	2.66
Gly	0.43	0.72	0.65	0.66	0.59	0.60
Pro	2.02	3.04	1.22	1.22	1.17	1.17
Ser	0.37	0.91	0.80	0.74	0.68	0.69
Tyr	0.48	0.69	0.63	0.63	0.53	0.53

^1^ Basal-2, corn–soybean basal diet. ^2^ J1: Heilongjiang Shenglong Industrial Co., Ltd. (Harbin, China); J2: Hei-longjiang Zhongke Green Biotechnology Co., Ltd. (Mudanjiang, China); N3: Henan Mengzhou Huaxing Co., Ltd. (Jiaozuo, China); X4: Xinjiang Bosheng Liquor Brewing Co., Ltd. (Bortala Mongolian Autonomous Prefecture, China); C5: Chifeng Ruiyang Chemical Co., Ltd. (Chifeng, China). J1-D1, J2-D2, N3-D3, X4-D4 and C5-D5 were test diets containing 30% corn DDGSs from 5 different sources. ^3^ GE, gross energy; CP, crude protein; NDF, neutral detergent fiber; ADF, acid detergent fiber; EE, ether extract; Ash, crude ash; AA, amino acid; Arg, arginine; His, histidine; Leu, leucine; Ile, Isoleucine; Lys, Lysine; Met, methionine; Phe, phenylalanine; Thr, threonine; Trp, tryptophan; Val, valine; Ala, alanine; Asp, aspartic; Cys, cystine; Gly, glycine; Pro, proline; Ser, serine; Tyr, tyrosine.

**Table 11:** Nutritional composition and CV of corn DDGSs samples (DM basis).

Item ^1^	Corn DDGSs ^2^					CV ^3^
	J1	J2	N3	X4	C5	
GE (MJ/kg)	19.56	20.33	22.23	20.12	22.15	5.3%
CP	28.42	28.54	29.99	26.23	29.36	4.9%
NDF	36.68	38.19	34.41	35.90	29.88	9.0%
ADF	10.72	11.00	10.38	10.97	9.09	6.9%
EE	3.92	11.47	12.00	9.98	12.15	35.8%
Ash	5.16	5.27	5.39	5.02	5.00	3.1%

^1^ GE, gross energy; CP, crude protein; NDF, neutral detergent fiber; ADF, acid detergent fiber; EE, ether extract; Ash, crude ash. ^2^ J1: Heilongjiang Shenglong Industrial Co., Ltd. (Harbin, China); J2: Hei-longjiang Zhongke Green Biotechnology Co., Ltd. (Mudanjiang, China); N3: Henan Mengzhou Huaxing Co., Ltd. (Jiaozuo, China); X4: Xinjiang Bosheng Liquor Brewing Co., Ltd. (Bortala Mongolian Autonomous Prefecture, Xinjiang Uygur Autonomous Region, China); C5: Chifeng Ruiyang Chemical Co., Ltd. (Chifeng, China). ^3^ CV, coefficients of variation.

**Table 12:** Effects of different diets for pregnant sows on nutrient digestibility and nitrogen balance.

Item ^1^	Basal-2 ^2^	Test Diets ^3^					SEM	*p* Value
		J1-D1	J2-D2	N3-D3	X4-D4	C5-D5		
DM intake (kg/d)	2.22	2.13	2.09	2.22	2.30	2.13	0.03	0.32
ATTD (%)								
DM	90.70 ^a^	90.76 ^a^	86.50 ^b^	90.47 ^a^	85.88 ^b^	89.53 ^a^	0.35	<0.01
OM	92.89 ^a^	92.63 ^a^	88.91 ^b^	92.34 ^a^	88.11 ^b^	91.61 ^a^	0.29	<0.01
GE	90.44 ^a^	90.73 ^a^	85.71 ^b^	90.21 ^a^	85.03 ^b^	88.99 ^a^	0.35	<0.01
CP	84.29 ^b^	87.77 ^a^	85.40 ^ab^	88.12 ^a^	83.37 ^b^	88.54 ^a^	0.44	<0.01
NDF	61.04 ^c^	75.84 ^ab^	66.80 ^c^	76.96 ^a^	68.32 ^bc^	77.69 ^a^	1.07	<0.01
ADF	74.16 ^b^	82.14 ^a^	76.44 ^ab^	82.19 ^a^	65.57 ^c^	74.36 ^b^	0.81	<0.01
EE	76.04 ^ab^	73.40 ^abc^	64.98 ^c^	79.42 ^a^	67.49 ^bc^	78.98 ^a^	1.20	<0.01
Nitrogen balance (g/d)								
Intake	40.83 ^b^	60.60 ^a^	56.31 ^a^	61.72 ^a^	59.82 ^a^	56.67 ^a^	0.75	<0.01
Feces output	6.56	7.43	9.58	8.12	8.11	6.51	0.50	0.50
Urine output	18.21	27.93	20.67	33.27	25.92	18.73	2.57	0.50
Retention	16.06	25.24	26.06	20.33	25.80	31.44	2.80	0.70

^1^ ATTD, apparent total tract digestibility; DM, dry matter; OM, organic matter; GE, gross energy; CP, crude protein; NDF, neutral detergent fiber; ADF, acid detergent fiber; EE, ether extract; DE, digestible energy; ME; metabolizable energy; SEM, standard error of the mean. Different lowercase letters on the shoulder of peer data indicate significant differences (*p* < 0.05), while the same or no letters indicate no significant differences (*p* > 0.05). ^2^ Basal-2, corn–soybean basal diet. ^3^ J1: Heilongjiang Shenglong Industrial Co., Ltd. (Harbin, Heilongjiang); J2: Hei-longjiang Zhongke Green Biotechnology Co., Ltd. (Mudanjiang, China); N3: Henan Mengzhou Huaxing Co., Ltd. (Jiaozuo, China); X4: Xinjiang Bosheng Liquor Brewing Co., Ltd. (Bortala Mongolian Autonomous Prefecture, China); C5: Chifeng Ruiyang Chemical Co., Ltd. (Chifeng, China). Different lowercase letters on the shoulder of peer data indicate significant differences (*p* < 0.05), while the same or no letters indicate no significant differences (*p* > 0.05). J1-D1, J2-D2, N3-D3, X4-D4 and C5-D5 were test diets containing 30% corn DDGSs from 5 different sources.

**Table 13:** Effect of diets on energy balance and available energy value for pregnant sows.

Item	Basal-2	Test Diets					SEM	*p* Value
		J1-D1	J2-D2	N3-D3	X4-D4	C5-D5		
Body weight changes (kg)	−10.9	−11.5	−4.3	−6.4	−8.8	−12.5	1.47	0.41
Energy balance (kJ/kg BW^0.75^/d)								
ME intake	475.60	421.18	439.17	498.72	485.51	473.26	10.03	0.24
THP	483.20	457.17	479.54	455.53	460.78	464.05	11.48	0.97
FHP	430.67	395.58	458.55	408.20	430.53	402.96	11.38	0.66
REP	39.29	62.23	67.92	51.70	64.53	79.92	7.20	0.68
REL	−39.30	−62.27	−67.97	−51.65	−64.50	−79.92	7.20	0.68
RQ								
Fed state	0.93	0.91	0.86	0.89	0.93	0.92	0.01	0.73
Fasted state	0.85	0.89	0.82	0.82	0.86	0.90	0.02	0.76
Energy values (MJ/kg DM)								
DE	14.01	14.24	13.30	14.32	14.04	14.30	0.12	0.18
ME	12.93	12.26	11.93	12.84	12.76	12.72	0.18	0.63
NE	11.51	10.55	11.30	11.57	11.97	11.07	0.27	0.74
Energy utilization (%)								
UE/DE	6.71	14.39	8.63	9.10	7.86	9.90	1.06	0.40
CH_4_E/DE	1.02	1.61	1.41	1.24	1.19	1.24	0.06	0.15
ME/DE	92.28	86.11	89.86	89.66	90.95	88.87	1.09	0.68
NE/ME	88.75	86.22	94.54	90.24	93.73	87.34	1.67	0.65

J1: Heilongjiang Shenglong Industrial Co., Ltd. (Harbin, China); J2: Hei-longjiang Zhongke Green Biotechnology Co., Ltd. (Mudanjiang, China); N3: Henan Mengzhou Huaxing Co., Ltd. (Jiaozuo, China); X4: Xinjiang Bosheng Liquor Brewing Co., Ltd. (Bortala Mongolian Autonomous Prefecture, China); C5: Chifeng Ruiyang Chemical Co., Ltd. (Chifeng, China). J1-D1, J2-D2, N3-D3, X4-D4 and C5-D5 were test diets containing 30% corn DDGSs from 5 different sources. THP, total heat production; FHP, fasting heat production; RE_P_, energy retention as protein; RE_L_, energy retention as lipid; RQ, respiratory quotient; DE, digestible energy; ME; metabolizable energy; NE, net energy; UE, urine energy; CH_4_E, CH_4_ energy; SEM, standard error of the mean.

**Table 14:** ATTD, energy conversion rates and energy values of different corn DDGSs in pregnant sows.

Item ^1^	Corn DDGSs ^2^					SEM	*p* Value
	J1	J2	N3	X4	C5		
ATTD (%)							
DM	87.54 ^a^	76.28 ^bc^	87.64 ^a^	74.13 ^c^	84.26 ^ab^	1.23	<0.01
OM	89.32 ^a^	79.19 ^b^	89.14 ^a^	76.47 ^b^	86.51 ^a^	1.00	<0.01
GE	88.10 ^a^	74.19 ^b^	87.35 ^a^	71.86 ^b^	82.80 ^a^	1.22	<0.01
CP	85.04 ^ab^	88.12 ^ab^	94.34 ^ab^	81.13 ^b^	96.84 ^a^	1.81	0.05
NDF	82.93 ^ab^	68.33 ^b^	86.94 ^ab^	76.32 ^bc^	93.28 ^a^	1.98	<0.01
ADF	94.96 ^a^	81.99 ^ab^	97.72 ^a^	50.90 ^c^	67.79 ^b^	1.40	<0.01
EE	58.46 ^ab^	38.04 ^b^	82.42 ^a^	46.66 ^b^	78.62 ^a^	3.49	<0.01
Energy value (MJ/kg DM)							
DE	16.06	16.88	18.07	15.58	17.52	0.36	0.18
ME	12.17	12.41	15.76	13.79	16.42	0.53	0.08
NE	8.76 ^c^	11.81 ^bc^	13.30 ^ab^	12.18 ^abc^	15.88 ^a^	0.55	0.02
Energy utilization (%)							
ME/DE	82.44	81.39	82.00	85.37	90.21	2.36	0.76
NE/ME	68.19	92.64	82.74	92.11	87.47	4.83	0.58

^1^ ATTD, apparent total tract digestibility; DM, dry matter; OM, organic matter; GE, gross energy; CP, crude protein; NDF, neutral detergent fiber; ADF, acid detergent fiber; EE, ether extract; DE, digestible energy; ME; metabolizable energy; NE, net energy; SEM, standard error of the mean. Different lowercase letters on the shoulder of peer data indicate significant differences (*p* < 0.05), while the same or no letters indicate no significant differences (*p* > 0.05). ^2^ J1: Heilongjiang Shenglong Industrial Co., Ltd. (Harbin, China); J2: Hei-longjiang Zhongke Green Biotechnology Co., Ltd. (Mudanjiang, China); N3: Henan Mengzhou Huaxing Co., Ltd. (Jiaozuo, China); X4: Xinjiang Bosheng Liquor Brewing Co., Ltd. (Bortala Mongolian Autonomous Prefecture, China); C5: Chifeng Ruiyang Chemical Co., Ltd. (Chifeng, China).

**Table 15:** Correlation analysis of chemical composition and energy value of five corn DDGS samples.

Item	DE	ME	NE	GE	CP	NDF	ADF	HC	EE	Ash
DE	1.00									
ME	0.69	1.00								
NE	0.62	0.89	1.00							
GE	0.85	0.81	0.57	1.00						
CP	0.92 *	0.48	0.35	0.85	1.00					
NDF	−0.50	−0.89 *	−0.81	−0.76	−0.40	1.00				
ADF	−0.57	−0.80	−0.76	−0.79	−0.54	0.96 *	1.00			
HC	−0.47	−0.91 *	−0.81	−0.74	−0.35	0.99 *	0.94 *	1.00		
EE	0.62	0.66	0.86	0.32	0.28	−0.40	−0.36	−0.41	1.00	
Ash	0.53	−0.10	−0.22	0.22	0.56	0.43	0.36	0.45	0.12	1.00

DE, digestible energy; ME; metabolizable energy; NE, net energy; GE, gross energy; CP, crude protein; NDF, neutral detergent fiber; ADF, acid detergent fiber; HC, hemicellulose; EE, ether extract; Ash, crude ash. * *p* < 0.05.

**Table 16:** Prediction equations for available energy values (MJ/kg) from chemical composition of corn DDGSs (% or MJ/kg dry matter) in pregnant sows.

Equations for Corn DDGSs (n = 5)	R^2^	RMSE	*p* Value
DE = −0.86 + 0.58 CP + 0.12 EE	0.98	0.16	0.01
ME = 27.07 + 0.19 EE − 0.61 HC	0.84	0.78	0.08
NE = 23.45 − 0.45 NDF + 0.47 EE	0.98	0.30	0.01

DE, digestible energy; ME; metabolizable energy; NE, net energy; GE, gross energy; CP, crude protein; EE, ether extract; HC, hemicellulose; RMSE, root mean square error.

**Table 17:** Comparison of determined and predicted available energy of 5 types of corn DDGSs (MJ/kg, dry matter basis).

Item	DE of Ingredient			ME of Ingredient			NE of Ingredient		
	Determined	Predicted	|RE|	Determined	Predicted	|RE|	Determined	Predicted	|RE|
J1	16.06	16.07	0.07%	12.17	12.12	0.39%	8.76	8.80	0.51%
J2	16.88	17.02	0.85%	12.41	12.83	3.41%	11.81	11.70	0.95%
N3	18.07	17.93	0.80%	15.76	14.85	5.81%	13.30	13.65	2.62%
X4	15.58	15.51	0.43%	13.79	13.91	0.88%	12.18	12.02	1.29%
C5	17.52	17.58	0.33%	16.42	16.84	2.53%	15.88	15.76	0.78%

J1: Heilongjiang Shenglong Industrial Co., Ltd. (Harbin, China); J2: Hei-longjiang Zhongke Green Biotechnology Co., Ltd. (Mudanjiang, China); N3: Henan Mengzhou Huaxing Co., Ltd. (Jiaozuo, China); X4: Xinjiang Bosheng Liquor Brewing Co., Ltd. (Bortala Mongolian Autonomous Prefecture, China); C5: Chifeng Ruiyang Chemical Co., Ltd. (Chifeng, China). DE, digestible energy; ME; metabolizable energy; NE, net energy; RE, relative error.

## Data Availability

Data is contained within the article.
